# Caring for our cancer patients in the wake of COVID-19

**DOI:** 10.1038/s41416-020-0843-5

**Published:** 2020-04-17

**Authors:** Karim Hussien El-Shakankery, Joanna Kefas, Shanthini Mary Crusz

**Affiliations:** 1 Department of Medicine, Whipps Cross University Hospital, London, UK; 2 Department of Medical Oncology, St Bartholomew’s Hospital, London, UK

**Keywords:** Chemotherapy, Cancer immunotherapy

## Abstract

In response to our current global pandemic, unprecedented healthcare changes may have significant consequences for cancer patients in the United Kingdom. We explore why cancer patients may be more susceptible to severe infection and complications, highlighting various interventions that may help to ensure continuity of care in this unique cohort.

## Main

As the world continues to brace itself for the ongoing wave of the new coronavirus, severe acute respiratory syndrome coronavirus 2 (SARS-CoV-2, COVID-19), healthcare systems globally are struggling more than ever before. In response to the global pandemic, the United Kingdom’s National Health Service has implemented several interventions aiming to increase hospital capacity, including delaying elective operations and non-urgent radiological scans, and re-purposing clinical areas and infusion suites to form novel front-line wards. In addition, an announcement by the National Institute for Health Research has called for a pause in commencement of all new clinical trials in order to mobilise and maximise healthcare resources.^1^ Indeed, during this time, many of these changes will inevitably have adverse consequences on the day-to-day care of patients with cancer.

The potential to cause harm by COVID-19 is at least three-fold in oncology patients. Firstly, in the vulnerability to infection that both the malignancy itself and systemic anticancer therapies (SACT) will bring—much of which still remains unknown in the context of coronaviruses. Secondly, in the face of resource rationing, it is not known what effect delays in tumour resection surgeries and administration of SACTs will have on the long-term survival of this cohort; it is also not known for how long these delays will occur. Finally, those with cancer may well suffer under the challenge associated with who to allocate limited critical care and ventilated beds to, when compared with those without a significant co-morbidity such as cancer.

Although data on COVID-19 and malignancy remain limited at present, Liang et al. recently reported 18 cases of coronavirus infection in oncology patients (four who underwent chemotherapy or surgery within one month of infection, twelve on follow-up post tumour resection and two not known), noting that these patients were more likely to experience severe sequelae of COVID-19 infection (defined as intensive care admission, invasive ventilation or death).^2^

Of course, the myelosuppressive effects of chemotherapy and of metastatic malignancy are well understood, and it is reasonable to hypothesise that these patients will be more susceptible to severe infection and complications.^3^ Liang et al. estimate that the risk of developing severe events is significantly higher in patients with cancer, with a hazard ratio of 3.56, but we must be mindful that this is based on a small patient number (*n* = 18) and a heterogeneous population.^2^ Furthermore, some chemotherapy agents and immune-checkpoint inhibitors (ICI) can themselves contribute to or cause pneumonitis.^4,5^ Thus, managing COVID-19 in these patients could be challenging and requires a multidisciplinary approach due to the difficulties in distinguishing the contributions of SACT versus COVID-19.

In addition, the effect that novel anticancer treatments (such as ICI and targeted therapies) will have on the virulence and/or severity of COVID-19 has yet to be established. There is sparse literature commenting on the role of any other existing coronavirus strains and their interaction with these agents. However, as treatments such as ICI manipulate the immune system in various ways, it should be considered that their use could predispose to atypical manifestations of the coronavirus.^6^

Lessons learned from previous outbreaks, such as the 2012 Middle-East respiratory syndrome coronavirus (MERS-CoV) and the 2003 severe acute respiratory syndrome (SARS-CoV), have helped shape current guidance for COVID-19, but there are little data on how these infections affected cancer patients. Although both SARS and MERS largely pre-dated targeted therapies and ICI, there are a few studies specifically investigating the effect of chemotherapy during these times. For example, Chen et al. reported that of a cohort of 79 patients with non-small-cell lung cancer, none of them were documented to have contracted SARS, five were possible cases (proven negative) and during that time only ten of 373 chemotherapy sessions were delayed.^7^

The current global COVID-19 pandemic is unprecedented and continues to evolve. As such, how best to treat and support our cancer patients remains uncertain. New, comprehensive national guidance has assisted us to amend our practice accordingly during these challenging times, and there are several changes we can implement to ease the impact of this disease on our unique patient cohort^8^:

- Limit exposure
  - Virtual outpatient clinics
  - Virtual assessments of any possible symptoms prior to scheduled chemotherapy, with delayed treatment for those patients who have possible COVID-19 symptoms
  - Encouraging hand-washing and social distancing
  - Restrict visiting on inpatient wards

- Rationalise treatments
  - Prioritise SACT to patient groups who will have most benefit, e.g., in the neoadjuvant and adjuvant ‘curative’ setting
  - Consideration of treatment delays, especially in ‘high risk’ patients such as those with established cardiovascular comorbidities^8,9^
  - Prioritisation and rationalisation of surgeries based on urgency, symptoms and possibility of cure of cancer, also mindful of the need for subsequent post-operative critical care beds

- Limit morbidity
  - Consideration of increased use of prophylactic granulocyte-colony-stimulating factor alongside chemotherapy regimens to minimise neutropenic durations
  - Early identification of infection with on-the-door triage/assessments in those with fevers and symptoms
  - Delaying all treatments in COVID-positive or query patients, as this would enter an unknown field in which we do not fully understand the consequences
  - Ensure that patients are fully vaccinated (especially against influenza) to help rule out differential diagnoses in patients with possible respiratory infections
  - Provision of oncological support in decision-making for admitted COVID-19 cancer patients

With interventions such as those listed above, it is hoped that we can limit the impact of COVID-19 on our cancer patient population and not succumb to its ‘distraction effect’ in delivering their care.^10^ Despite our best efforts, there will be further challenges awaiting us as fallout to changes in standard practice once we have recovered from the pandemic. But for now, we must focus on the immediacy of protecting our cancer patients as best as we can.

## Data Availability

No data were collected for use in this paper. Any data highlighted in this paper from previous studies have been referenced.
